# Renoprotective Effect of Dipeptidyl Peptidase-4 Inhibitors in Patients with Type 2 Diabetes Mellitus

**DOI:** 10.3389/fphar.2017.00835

**Published:** 2017-11-15

**Authors:** Hiroki Esaki, Tomoya Tachi, Chitoshi Goto, Ikuto Sugita, Yuta Kanematsu, Aki Yoshida, Kosuke Saito, Yoshihiro Noguchi, Yuki Ohno, Satoshi Aoyama, Masahiro Yasuda, Takashi Mizui, Masumi Yamamura, Hitomi Teramachi

**Affiliations:** ^1^Laboratory of Clinical Pharmacy, Gifu Pharmaceutical University, Gifu, Japan; ^2^Department of Pharmacy, Ichinomiya Municipal Hospital, Ichinomiya, Japan; ^3^Department of Pharmacy, Gifu Municipal Hospital, Gifu, Japan; ^4^Laboratory of Community Healthcare Pharmacy, Gifu Pharmaceutical University, Gifu, Japan

**Keywords:** dipeptidyl peptidase-4, type 2 diabetes mellitus, renal function, multiple logistic regression, Cox model

## Abstract

Diabetic nephropathy is one of three major complications of diabetes mellitus, often leading to chronic renal failure requiring dialysis. Recently developed dipeptidyl peptidase-4 (DPP-4) inhibitors may exhibit renoprotective effects in addition to antihyperglycemic effects. In this study, we retrospectively investigated temporal changes in the renal function index of patients with type 2 diabetes mellitus (DM) and examined the influence of DPP-4 inhibitors on renal function. Patients with type 2 DM (>18 years old) prescribed hypoglycemic agents at Gifu Municipal Hospital for ≥3 months between March 2010 and April 2014 were included in the study. Renal function was evaluated as estimated the decline in 12-month glomerular filtration rate from the baseline in patients receiving and not receiving DPP-4 inhibitors. Patient data from the DPP-4 inhibitor-treated (501 patients, 58.6%) and untreated (354, 41.4%) groups were analyzed using multiple logistic regression analysis, as well as Cox proportional-hazards regression analysis (616, 55.6% and 491, 44.4%, for DPP-4 inhibitors-treated and untreated groups). Multiple logistic regression analysis indicated that DPP-4 inhibitors significantly lowered the estimated glomerular filtration rate (eGFR) decline [20% over 12 months; odds ratio (OR), 0.626; 95% confidence interval [CI], 0.409–0.958; *P* = 0.031]. Similar results were obtained using Cox proportional-hazards regression analysis (hazard ratio [HR], 0.707; 95% CI, 0.572–0.874; *P* = 0.001). These findings suggest that DPP-4 inhibitors suppress the decrease of estimated glomerular filtration rate in patients with type 2 DM and show a renoprotective effect.

## Introduction

Recently developed dipeptidyl peptidase-4 (DPP-4) inhibitors enhance the function of endogenous incretins by selectively inhibiting incretin-degrading enzyme DPP-4 (Drucker and Nauck, 2006). Incretins are gut hormones secreted after food intake and include glucagon-like peptide 1 and glucose-dependent insulinotropic polypeptide (Nauck et al., 2009). Incretins decrease blood glucose levels by promoting the secretion of insulin from pancreatic β-cells in a glucose concentration-dependent manner an inhibiting secretion of glucagon from α-cells (Idris and Donnelly, 2007; Holst et al., 2016). According to recent reports, incretin receptors are present in various organs, and incretin effects are not limited to the pancreas (Kim and Samson, 2014). The renoprotective effects of incretins are independent of their hypoglycemic effects and likely mediated by the suppression of sodium reabsorption, anti-oxidative, and anti-inflammatory effects on the renal tubules (Hirata et al., 2009; Kodera et al., 2011; Mima et al., 2012; Joo et al., 2013; Fujita et al., 2014; Vallon and Docherty, 2014; Duvnjak and Blaslov, 2016).

Medical costs for diabetes mellitus (DM) treatment and its associated complications have reached 37 billion dollars in Japan and 825 billion dollars globally in 2014 (NCD Risk Factor Collaboration, 2016). Increasing incidence of DM suggests that treatment costs will continue to increase. Diabetic nephropathy is one of three major DM complications. Since 1998, diabetic nephropathy has been the primary disease resulting in dialysis in Japan (http://docs.jsdt.or.jp/overview/, accessed 1st September, 2016), and is currently the primary cause of end-stage renal disease (ESRD) in the US (https://www.usrds.org/2015/view/Default.aspx, accessed 1st September, 2016). Degradation of renal function is a risk factor for ESRD, the occurrence of cardiovascular events, and death (So et al., 2006; Matsushita et al., 2010; Hallan et al., 2012; Coresh et al., 2014). In addition, ESRD requires dialysis treatments, which decreases the quality of life of patients (Feroze et al., 2011; Md Yusop et al., 2013; Saad et al., 2015). These data emphasize the necessity of preventing the development and progression of diabetic nephropathy and importance of delaying the degradation of renal function.

The effects of DPP-4 inhibitors on renal function have been previously described. The coadministration of linagliptin, a DPP-4 inhibitor, drastically lowered albuminuria in patients with type 2 DM with renal disorders treated with renin-angiotensin-aldosterone system inhibitors, compared to the placebo (Groop et al., 2013), whereas alogliptin, another DPP-4 inhibitor, lowered albuminuria in patients with type 2 DM with early-stage nephropathy treated with angiotensin II receptor blockers (ARBs) (Fujita et al., 2014). These studies demonstrated the effects of additional treatment with DPP-4 inhibitors, whereas no effects of DPP-4 inhibitors monotherapy were examined. Most studies focusing on DPP-4 inhibitors and renal function did not indicate any renoprotective effects of DPP-4 inhibitors; however, these reports clarified the safety and tolerability of DPP-4 inhibitors in patients with renal dysfunction (Chan et al., 2008; Kothny et al., 2012; Groop et al., 2014).

Linagliptin can be used in patients with renal dysfunction without dose adjustment because it is excreted in the bile. Linagliptin reduced the decrease in estimated glomerular filtration rate (eGFR) in patients with type 2 DM with severe renal dysfunction, compared to those administered the placebo (McGill et al., 2013). Linagliptin monotherapy was reported to inhibit the degradation of renal function; however, the effect was limited to patients with severe renal dysfunction. Renoprotective effects of other DPP-4 inhibitors have not been extensively investigated.

In this study, we retrospectively analyzed the data of patients with type 2 DM treated with hypoglycemic agents to clarify the effects of DPP-4 inhibitor monotherapy on renal function. We assessed the renoprotective effects of DPP-4 inhibitors using multiple classification methods to avoid confounding biases.

## Methods

Patients with Type 2 DM (>18 years old) who were prescribed hypoglycemic agents at Gifu Municipal Hospital for ≥3 months between March 2010 and April 2014 were retrospectively considered for inclusion in this study.

Patient data (age, sex, body height, body weight, systolic blood pressure (BP), diastolic BP, laboratory data, relevant medical history, and concurrent medications) from electronic health records were analyzed. Patient laboratory data included serum albumin, blood urea nitrogen, serum creatinine, uric acid, triglyceride, high-density lipoprotein (HDL) cholesterol, low-density lipoprotein (LDL) cholesterol, serum sodium, serum potassium, serum chloride, and hemoglobin A1c (HbA1c) at baseline and 3, 6, and 12 months post-treatment. We collected medical history records of hypertension, dyslipidemia, and hyperuricemia, diseases affecting renal function, and duration of treatment with hypoglycemic or potentially nephrotoxic agents (Perazella, 2009). Patients with a history of dialysis treatment or renal transplantation prescribed DPP-4 inhibitors at other medical institutions, prescribed glucagon-like peptide 1 receptor agonists, and those continuing treatment with hypoglycemic agents prescribed before the survey period for this study were excluded. Patients with available serum creatinine level data at baseline and 12 months post-treatment were included in the multiple logistic regression analysis, whereas patients with serum creatinine data at baseline and 3, 6, or 12 months post-treatment were included in the Cox proportional-hazards regression analysis.

We assessed the renal function using eGFR decline. The eGFR was calculated using the following formula: eGFR = 194 × serum creatinine^−1.094^ × age^−0.287^ (mL·min^−1^·1.73 m^(2)−1^), with eGFR adjusted to eGFR × 0.739 for female patients (Matsuo et al., 2009). We calculated the eGFR decline after 3 months using the following formula: (eGFR_baseline_−eGFR_3months_)/eGFR_baseline_ × 100. The eGFR decline after 6 and 12 months was defined analogously.

We used the IBM statistical package for the social sciences (SPSS) software, version 24.0J (Armonk, NY, USA) for the statistical analysis. Unpaired *t*-test, χ^2^ test and Fisher's exact test were used to analyze the differences between the DPP-4 inhibitor-treated and untreated groups. Multiple logistic regression analysis and Cox regression were performed with eGFR decline of >10, >20, or >30% as the dependent variable and “DPP-4 inhibitors,” “age ≥ 65 years” (Yamagata et al., 2007; Kazancioglu, 2013), “male sex” (Kazancioglu, 2013; de Hauteclocque et al., 2014), “hypertension” (Rossing et al., 2004; Higashikuni et al., 2008), “dyslipidemia” (Muntner et al., 2000; Schaeffner et al., 2003; Yamagata et al., 2007), “hyperuricemia” (Yamagata et al., 2007; Li et al., 2014), “angiotensin-converting enzyme inhibitors (ACEI)/ARBs” (Lewis et al., 1993, 2001; Brenner et al., 2001; Zhu et al., 2008; Ruggenenti et al., 2010), “statins” (Lee et al., 2002; Tonelli et al., 2005), and “nephrotoxic agents” (Perazella, 2009) as independent variables to avoid confounding biases. *P* < 0.05 were considered statistically significant.

This study was approved by the Ethical Review Board of Gifu Municipal Hospital (approval number 203) and the Bioethics Committee of Gifu Pharmaceutical University (approval number Hei27-14). Furthermore, the opt-out consent approach approved by both ethical committees was used in this study. Based on the Ethical Guidelines for Medical and Health Research Involving Human Subjects (Ministry of Health, Labour and Welfare of Japan), obtaining written informed consent from patients was not compulsory because this was a pharmacoepidemiological study that did not require any interventions or interactions with patients because it used pre-existing material and information.

## Results

### Patient selection

**Figure 1:**
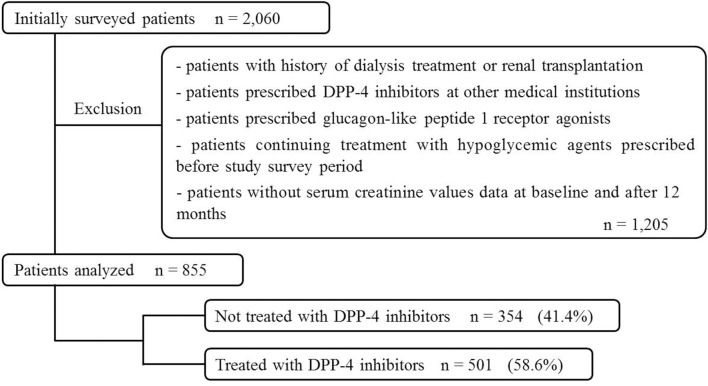
Patient selection (multiple logistic regression analysis).

Of the 2,060 patients initially screened for study inclusion, 855 were included in the multiple logistic regression analysis and categorized into DPP-4 inhibitor-untreated (354 patients, 41.4%) and DPP-4 inhibitor-treated group (501, 58.6%; Figure 1).

**Figure 2:**
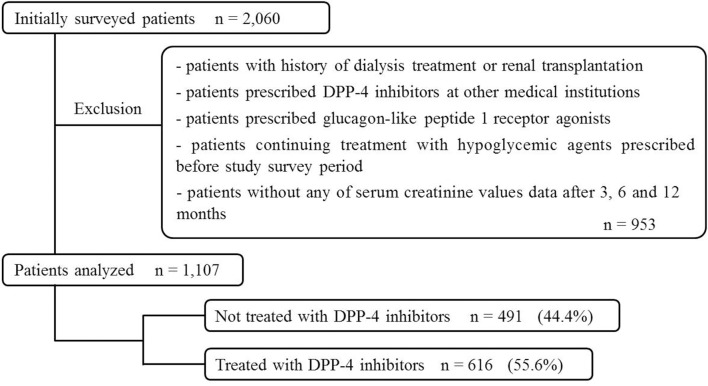
Patient selection (Cox proportional-hazards regression analysis).

Similarly, 1,107 patients were included in the Cox proportional-hazards regression analysis (491 patients, 44.4%, DPP-4 untreated group; 616 patients, 55.6%, DPP-4-treated group, Figure 2).

### Patient baseline characteristics

The baseline characteristics of patients included in the multiple logistic regression analysis are shown in Table 1. The average age of patients was 64.5 ± 12.0 years (mean ± SD), and 53.9% of the patients were >65 years old (64.4% men). Hypertension, dyslipidemia, and hyperuricemia were reported in 61.1, 53.8, and 10.1% of the patients, respectively. Patients treated with DPP-4 inhibitors showed significantly higher systolic blood pressure (BP), diastolic BP, serum albumin, dyslipidemia, and eGFR reduction rate (baseline to 12 months) than the untreated patients did. Baseline characteristics of patients included in the Cox proportional-hazards regression analysis are shown in Table 2. The average patient age was 64.4 ± 12.4 years, and 54.2% of the patients were >65 years old (64.0% men). Hypertension, dyslipidemia, and hyperuricemia were reported in 60.4, 49.8, and 9.8% of the patients. Patients treated with DPP-4 inhibitors showed significantly higher systolic BP, diastolic BP, serum albumin, serum sodium, dyslipidemia, and eGFR reduction rate (baseline to 3 and 12 months) than the DPP-4 inhibitor-untreated patients did.

**Table 1:** Patient baseline characteristics (multiple logistic regression analysis).

	**Overall (*n* = 855)**	**DPP-4 inhibitors-untreated group (*n* = 354)**	**DPP-4 inhibitors-treated group (*n* = 501)**	***P*-value**
Age (years)	64.5 ± 12.0	64.3 ± 12.4	64.7 ± 11.7	0.637
≥ 65 years [*n* (%)]	461 (53.9)	191 (54.0)	270 (53.9)	0.986
Male sex [*n* (%)]	551 (64.4)	234 (66.1)	317 (63.3)	0.395
Body height (cm)	160.7 ± 9.4 (*n* = 810)	160.5 ± 9.3 (*n* = 337)	160.9 ± 9.5 (*n* = 473)	0.619
Body weight (kg)	64.7 ± 14.9 (*n* = 829)	64.7 ± 15.2 (*n* = 347)	64.7 ± 14.7 (*n* = 482)	0.956
BMI (kg/m^2^)	24.9 ± 4.7 (*n* = 800)	24.9 ± 5.0 (*n* = 336)	24.9 ± 4.6 (*n* = 464)	0.974
Systolic BP (mmHg)	134.5 ± 20.3 (*n* = 622)	131.8 ± 18.8 (*n* = 247)	136.3 ± 21.0 (*n* = 375)	0.006^*^
Diastolic BP (mmHg)	77.3 ± 14.0 (*n* = 622)	75.1 ± 13.3 (*n* = 247)	78.7 ± 14.3 (*n* = 375)	0.002^*^
**LABORATORY FINDINGS**				
Serum albumin (g/dL)	4.1 ± 0.5 (*n* = 516)	4.1 ± 0.6 (*n* = 225)	4.2 ± 0.5 (*n* = 291)	0.021^*^
BUN (mg/dL)	16.4 ± 7.3 (*n* = 844)	16.8 ± 8.6 (*n* = 349)	16.2 ± 6.2 (*n* = 495)	0.276
Serum creatinine (mg/dL)	0.81 ± 0.38 (*n* = 855)	0.85 ± 0.47 (*n* = 354)	0.79 ± 0.30 (*n* = 501)	0.056
Uric acid (mg/dL)	5.5 ± 1.5 (*n* = 366)	5.6 ± 1.5 (*n* = 128)	5.5 ± 1.4 (*n* = 238)	0.298
Triglyceride (mg/dL)	168.3 ± 120.6 (*n* = 667)	171.8 ± 131.7 (*n* = 253)	166.2 ± 113.4 (*n* = 414)	0.564
HDL-cholesterol (mg/dL)	51.4 ± 15.1 (*n* = 648)	52 ± 15.9 (*n* = 240)	51 ± 14.6 (*n* = 408)	0.397
LDL-cholesterol (mg/dL)	111.9 ± 30.6 (*n* = 595)	115.7 ± 32.5 (*n* = 212)	109.8 ± 29.3 (*n* = 383)	0.023^*^
Serum sodium (mEq/L)	139.2 ± 3.1 (*n* = 677)	139 ± 3.3 (*n* = 268)	139.2 ± 2.9 (*n* = 409)	0.350
Serum potassium (mEq/L)	4.3 ± 0.5 (*n* = 679)	4.3 ± 0.5 (*n* = 270)	4.3 ± 0.4 (*n* = 409)	0.144
Serum chloride (mEq/L)	103.4 ± 3.5 (*n* = 672)	103.4 ± 3.7 (*n* = 265)	103.3 ± 3.3 (*n* = 407)	0.651
HbA1c (%)	8.0 ± 1.7 (*n* = 802)	8.1 ± 2.1 (*n* = 328)	8.0 ± 1.5 (*n* = 474)	0.500
eGFR (mL•min^−1^•1.73 m^(2)−1^)	76.6 ± 26.4	76.4 ± 28.4	76.7 ± 24.9	0.891
**eGFR REDUCTION RATE**				
Baseline to 12 months (%)	3.1 ± 16.5	4.7 ± 15.5	2.0 ± 17.1	0.020^*^
**eGFR [*n* (%)]**				
≥ 90 (mL•min^−1^•1.73 m^(2)−1^)	223 (26.1)	92 (26)	131 (26.1)	
60–90 (mL•min^−1^•1.73 m^(2)−1^)	427 (49.9)	180 (50.8)	247 (49.3)	
45–60 (mL•min^−1^•1.73 m^(2)−1^)	127 (14.9)	49 (13.8)	78 (15.6)	
30–45 (mL•min^−1^•1.73 m^(2)−1^)	44 (5.1)	13 (3.7)	31 (6.2)	
15–30 (mL•min^−1^•1.73 m^(2)−1^)	31 (3.6)	17 (4.8)	14 (2.8)	
<15 (mL•min^−1^•1.73 m^(2)−1^)	3 (0.4)	3 (0.8)	0 (0.0)	
**RELEVANT MEDICAL HISTORY**				
Hypertension [*n* (%)]	522 (61.1)	212 (59.9)	310 (61.9)	0.557
Dyslipidemia [*n* (%)]	460 (53.8)	173 (48.9)	287 (57.3)	0.015^*^
Hyperuricemia [*n* (%)]	86 (10.1)	40 (11.3)	46 (9.2)	0.311

*Data are expressed as mean ± standard deviation unless otherwise indicated. DPP-4, dipeptidyl peptidase-4; BMI, body mass index; BP, blood pressure; BUN, blood urea nitrogen; HDL, high-density lipoprotein; LDL, low-density lipoprotein; HbA1c, hemoglobin A1c; eGFR, estimated glomerular filtration rate*.

* *P < 0.05*.

**Table 2:** Patient baseline characteristics (Cox proportional-hazards regression analysis).

	**Overall (*n* = 1,107)**	**DPP-4 inhibitors-untreated group (*n* = 491)**	**DPP-4 inhibitors-treated group (*n* = 616)**	***P*-value**
Age (years)	64.4 ± 12.4	64.3 ± 12.9	64.5 ± 12.0	0.766
≥ 65 years [*n* (%)]	600 (54.2)	265 (54.0)	335 (54.4)	0.891
Male sex [*n* (%)]	709 (64.0)	318 (64.8)	391 (63.5)	0.656
Body height (cm)	160.5 ± 9.4 (*n* = 1,049)	160.3 ± 9.2 (*n* = 468)	160.8 ± 9.6 (*n* = 581)	0.391
Body weight (kg)	64.7 ± 15.7 (*n* = 1,071)	64.7 ± 16.0 (*n* = 478)	64.7 ± 15.5 (*n* = 593)	0.985
BMI (kg/m^2^)	25.0 ± 4.9 (*n* = 1,037)	25.0 ± 5.1 (*n* = 465)	25.0 ± 4.7 (*n* = 572)	0.928
Systolic BP (mmHg)	134.9 ± 20.6 (*n* = 779)	132.4 ± 19.8 (*n* = 329)	136.7 ± 21.0 (*n* = 450)	0.003^*^
Diastolic BP (mmHg)	77.8 ± 14.3 (*n* = 779)	75.6 ± 14.0 (*n* = 329)	79.4 ± 14.3 (*n* = 450)	<0.001^*^
**LABORATORY FINDINGS**				
Serum albumin (g/dL)	4.0 ± 0.6 (*n* = 677)	3.9 ± 0.7 (*n* = 316)	4.1 ± 0.6 (*n* = 361)	<0.001^*^
BUN (mg/dL)	16.3 ± 7.4 (*n* = 1,090)	16.6 ± 8.3 (*n* = 482)	16 ± 6.5 (*n* = 608)	0.192
Serum creatinine (mg/dL)	0.81 ± 0.41 (*n* = 1,107)	0.8 ± 0.40 (*n* = 491)	0.80 ± 0.40 (*n* = 616)	0.194
Uric acid (mg/dL)	5.5 ± 1.5 (*n* = 457)	5.5 ± 1.5 (*n* = 171)	5.6 ± 1.5 (*n* = 286)	0.444
Triglyceride (mg/dL)	170.5 ± 122 (*n* = 810)	171.6 ± 129.8 (*n* = 327)	169.7 ± 116.5 (*n* = 483)	0.832
HDL-cholesterol (mg/dL)	50.8 ± 15.0 (*n* = 786)	51.6 ± 15.9 (*n* = 309)	50.3 ± 14.3 (*n* = 477)	0.252
LDL-cholesterol (mg/dL)	112.5 ± 31.6 (*n* = 716)	115.6 ± 32.3 (*n* = 271)	110.6 ± 31.0 (*n* = 445)	0.040^*^
Serum sodium (mEq/L)	139.1 ± 3.3 (*n* = 886)	138.7 ± 3.6 (*n* = 380)	139.3 ± 2.9 (*n* = 506)	0.003^*^
Serum potassium (mEq/L)	4.3 ± 0.5 (*n* = 887)	4.2 ± 0.5 (*n* = 381)	4.3 ± 0.4 (*n* = 506)	0.181
Serum chloride (mEq/L)	103.3 ± 3.6 (*n* = 876)	103.2 ± 3.7 (*n* = 373)	103.4 ± 3.4 (*n* = 503)	0.334
HbA1c (%)	8.1 ± 1.8 (*n* = 999)	8.2 ± 2.0 (*n* = 433)	8.1 ± 1.5 (*n* = 566)	0.352
eGFR (mL•min^−1^•1.73 m^(2)−1^)	77.7 ± 27.6	77.8 ± 29.2	77.6 ± 26.3	0.936
**eGFR REDUCTION RATE**				
Baseline to 3 months (%)	2.5 ± 15.9 (*n* = 1070)	3.7 ± 14.7 (*n* = 476)	1.6 ± 16.8 (*n* = 594)	0.034^*^
Baseline to 6 months (%)	2.6 ± 16.0 (*n* = 962)	3.8 ± 15.5 (*n* = 418)	1.8 ± 16.6 (*n* = 544)	0.052
Baseline to 12 months (%)	3.1 ± 16.5 (*n* = 855)	4.7 ± 15.5 (*n* = 354)	2.0 ± 17.1 (*n* = 501)	0.020^*^
**eGFR [*n* (%)]**				
≥ 90 (mL•min^−1^•1.73 m^(2)−1^)	310 (28.0)	143 (29.1)	167 (27.1)	
60–90 (mL•min^−1^•1.73 m^(2)−1^)	537 (48.5)	228 (46.4)	309 (50.2)	
45–60 (mL•min^−1^•1.73 m^(2)−1^)	156 (14.1)	72 (14.7)	84 (13.6)	
30–45 (mL•min^−1^•1.73 m^(2)−1^)	59 (5.3)	23 (4.7)	36 (5.8)	
15–30 (mL•min^−1^•1.73 m^(2)−1^)	40 (3.6)	21 (4.3)	19 (3.1)	
<15 (mL•min^−1^•1.73 m^(2)−1^)	5 (0.5)	4 (0.8)	1 (0.2)	
**RELEVANT MEDICAL HISTORY**				
Hypertension [*n* (%)]	669 (60.4)	288 (58.7)	381 (61.9)	0.280
Dyslipidemia [*n* (%)]	551 (49.8)	225 (45.8)	326 (52.9)	0.019^*^
Hyperuricemia [*n* (%)]	108 (9.8)	55 (11.2)	53 (8.6)	0.148

*Data are expressed as mean ± standard deviation unless otherwise indicated. DPP-4, dipeptidyl peptidase-4; BMI, body mass index; BP, blood pressure; BUN, blood urea nitrogen; HDL, high-density lipoprotein; LDL, low-density lipoprotein; HbA1c, hemoglobin A1c; eGFR, estimated glomerular filtration rate*.

* *P < 0.05*.

In both analyses, DPP-4 inhibitor-treated patients displayed significantly lower LDL-cholesterol levels than the untreated patients did, while no differences were observed in the HbA1c and eGFR.

### Concurrent patient medications

The concurrent medications taken by patients included in the multiple logistic regression analysis are shown in Table 3. Patients not treated with DPP-4 inhibitors were most commonly prescribed α-glucosidase inhibitors (153 patients, 43.2%). The most commonly prescribed DPP-4 inhibitors were sitagliptin (308, 61.5%), vildagliptin (130, 25.9%), alogliptin (37, 7.4%), linagliptin (18, 3.6%), and teneligliptin (8, 1.6%). Biguanides were the most commonly prescribed among other hypoglycemic agents (146, 29.1%). DPP-4 inhibitor untreated patients showed significantly higher usage of almost all agents except sulfonylureas than the DPP-4 inhibitor-treated patients did.

**Table 3:** Patient concurrent medication (multiple logistic regression analysis).

	**DPP-4 inhibitors-untreated group (*n* = 354)**	**DPP-4 inhibitors-treated group (*n* = 501)**	***P*-value**
**ANTIHYPERGLYCEMIC AGENTS**			
DPP-4 inhibitors			
Sitagliptin [*n* (%)]		308 (61.5)	
Vildagliptin [*n* (%)]		130 (25.9)	
Alogliptin [*n* (%)]		37 (7.4)	
Linagliptin [*n* (%)]		18 (3.6)	
Teneligliptin [*n* (%)]		8 (1.6)	
Biguanides [*n* (%)]	134 (37.9)	146 (29.1)	0.008^*^
Sulfonylureas [*n* (%)]	83 (23.4)	120 (24.0)	0.871
α-Glucosidase inhibitors [*n* (%)]	153 (43.2)	114 (22.8)	<0.001^*^
Insulin [*n* (%)]	118 (33.3)	83 (16.6)	<0.001^*^
Thiazolidines [*n* (%)]	62 (17.5)	53 (10.6)	0.004^*^
Glinides [*n* (%)]	34 (9.6)	3 (0.6)	<0.001^*^
**OTHER COMBINATION DRUG**			
ACEI/ARBs [*n* (%)]	119 (33.6)	166 (33.1)	
Statins [*n* (%)]	102 (28.8)	169 (33.7)	
Nephrotoxic agents [*n* (%)]	197 (55.6)	283 (56.5)	

*DPP-4, dipeptidyl peptidase-4; ACEI, angiotensin-converting enzyme inhibitors; ARBs, angiotensin II receptor blockers*.

* *P < 0.05*.

The analysis of concurrent medications used by patients included in the Cox regression is shown in Table 4. Patients not treated with DPP-4 inhibitors were most commonly treated with α-glucosidase inhibitors (196, 39.9%). Patients were prescribed the following DPP-4 inhibitors: sitagliptin (378, 61.4%), vildagliptin (158, 25.6%), alogliptin (46, 7.5%), linagliptin (23, 3.7%), and teneligliptin (11, 1.8%), whereas biguanides were the most commonly prescribed among other hypoglycemic agents (173, 28.1%). DPP-4 inhibitor untreated patients showed significantly higher usage of almost all agents except for sulfonylureas than the DPP-4 inhibitor-treated patients did.

**Table 4:** Patient concurrent medications (Cox proportional-hazards regression analysis).

	**DPP-4 inhibitors-untreated group (*n* = 491)**	**DPP-4 inhibitors-treated group (*n* = 616)**	***P*-value**
**Antihyperglycemic agents**			
DPP-4 inhibitors			
Sitagliptin [*n* (%)]		378 (61.4)	
Vildagliptin [*n* (%)]		158 (25.6)	
Alogliptin [*n* (%)]		46 (7.5)	
Linagliptin [*n* (%)]		23 (3.7)	
Teneligliptin [*n* (%)]		11 (1.8)	
Biguanides [*n* (%)]	182 (37.1)	173 (28.1)	0.002^*^
Sulfonylureas [*n* (%)]	105 (21.4)	145 (23.5)	0.426
α-Glucosidase inhibitors [*n* (%)]	196 (39.9)	127 (20.6)	<0.001^*^
Insulin [*n* (%)]	175 (35.6)	103 (16.7)	<0.001^*^
Thiazolidines [*n* (%)]	78 (15.9)	58 (9.4)	0.001^*^
Glinides [*n* (%)]	53 (10.8)	6 (1.0)	<0.001^*^
**OTHER COMBINATION DRUG**			
ACEI/ARBs [*n* (%)]	154 (31.4)	196 (31.8)	
Statins [*n* (%)]	123 (25.1)	190 (30.8)	
Nephrotoxic agents [*n* (%)]	263 (53.6)	335 (54.4)	

*DPP-4, dipeptidyl peptidase-4; ACEI, angiotensin-converting enzyme inhibitors; ARBs, angiotensin II receptor blockers*.

* *P < 0.05*.

### Multiple logistic regression analysis

**Figure 3:**
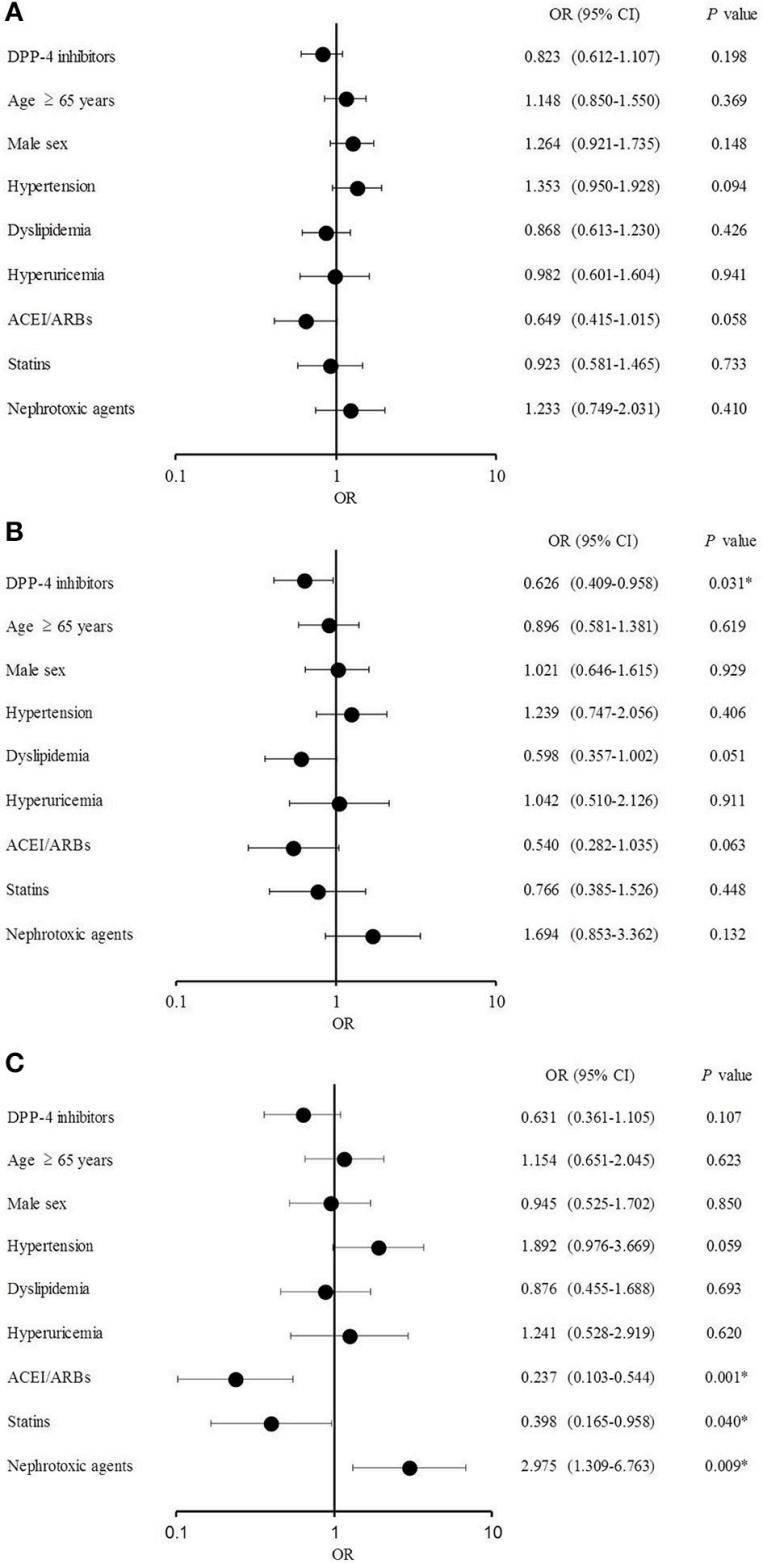
Multiple logistic regression analysis **(A)** Estimated glomerular filtration rate (eGFR) decline >10%. **(B)** eGFR decline >20%. **(C)** eGFR decline >30%.

The multiple logistic regression analysis results are shown in Figure 3. No significant difference was observed between the groups in the analysis of eGFR decline >10% over 12 months (Figure 3A), whereas DPP-4 inhibitors significantly reduced the risk of eGFR decline >20% [odds ratio (OR), 0.626; 95% confidence interval (CI), 0.409–0.958; *P* = 0.031; Figure 3B]. No significant difference was observed in effects of DPP-4 inhibitors on eGFR decline >30% over 12 months, the risk of which was significantly lowered by ACEI/ARBs (OR, 0.237; 95% CI, 0.103–0.544; *P* = 0.001) and statins (OR, 0.398; 95% CI, 0.165–0.958; *P* = 0.040) and increased by nephrotoxic agents (OR, 2.975; 95% CI, 1.309–6.763; *P* = 0.009; Figure 3C).

### Cox proportional-hazards regression analysis

**Figure 4:**
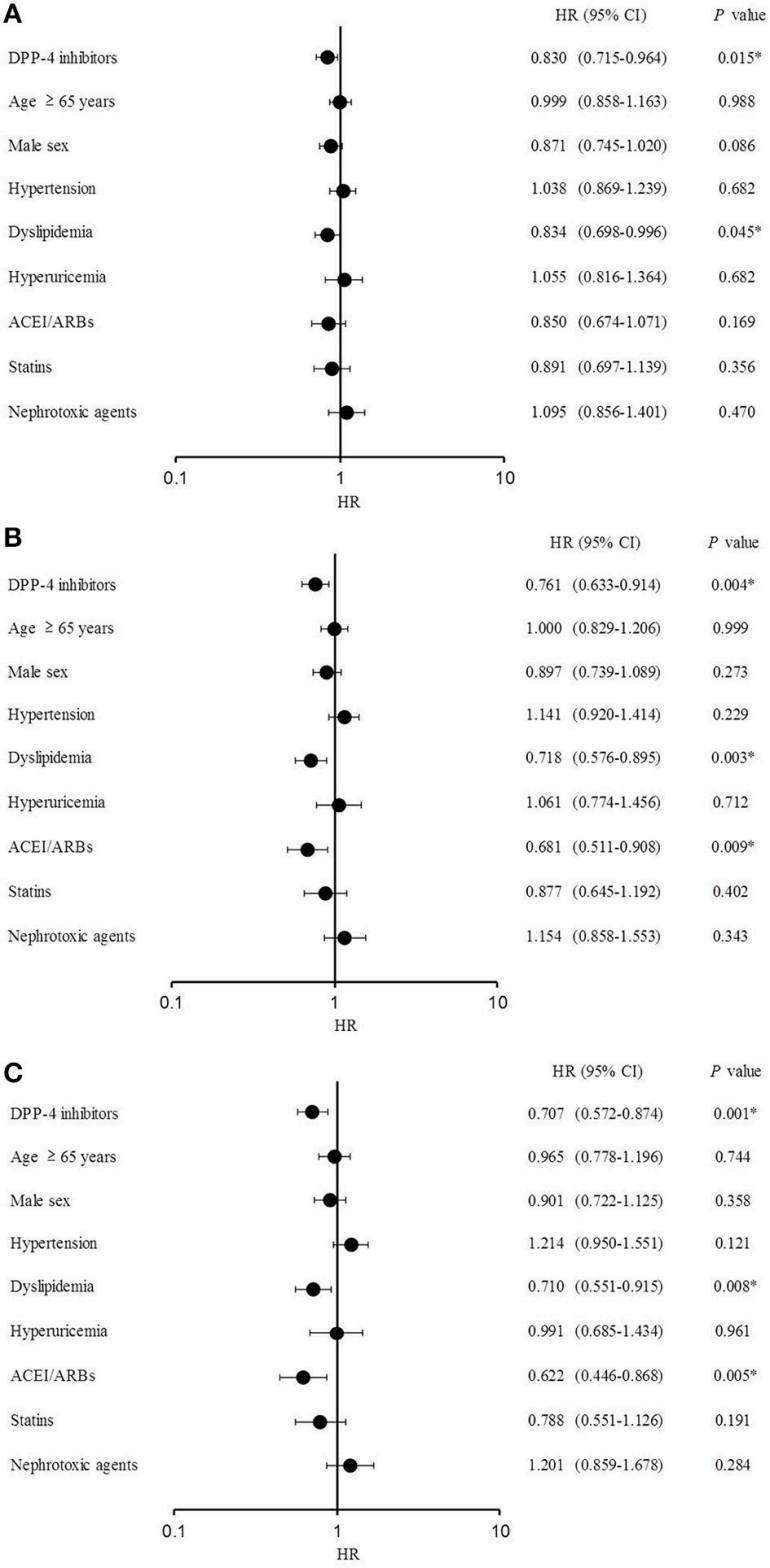
Cox proportional-hazards regression analysis. (**A)** Estimated glomerular filtration rate (eGFR) decline >10%. **(B)** eGFR decline >20%. **(C)** eGFR decline >30%.

The Cox proportional-hazards regression analysis results are shown in Figure 4. DPP-4 inhibitors significantly lowered the risk of eGFR decline > 10% [hazard ratio (HR), 0.830; 95% CI, 0.715–0.964; *P* = 0.015], as well as the occurrence of dyslipidemia (HR, 0.834; 95% CI, 0.698–0.996; *P* = 0.045). DPP-4 inhibitors (HR, 0.761; 95% CI, 0.633–0.914; *P* = 0.004), dyslipidemia (HR, 0.718; 95% CI, 0.576–0.895; *P* = 0.003), and ACEI/ARBs (HR, 0.681; 95% CI, 0.511–0.908; *P* = 0.009) significantly reduced the risk of eGFR decline >20% (Figure 4B). Similarly, DPP-4 inhibitors (HR, 0.707; 95% CI, 0.572–0.874; *P* = 0.001), dyslipidemia (HR, 0.710; 95% CI, 0.551–0.915; *P* = 0.008), and ACEI/ARBs (HR, 0.622; 95% CI, 0.446–0.868; *P* = 0.005) reduced the risk of eGFR decline >30% (Figure 4C).

## Discussion

In this study, the effects of DPP-4 inhibitors on renal function in patients with type 2 DM were examined using multivariate analysis. The OR of the DPP-4 inhibitors was significantly lower at eGFR decline >20% after 12 months than it was at baseline, suggesting that DPP-4 inhibitors suppressed the deterioration of renal function. Furthermore, the ORs of ACEI/ARBs and statins were significantly lower at eGFR decline >30% after 12 months than at baseline, whereas the OR of nephrotoxic agents was significantly higher, suggesting that ACEI/ARBs and statins suppressed while nephrotoxic agents accelerated the decline in renal function under these conditions. These results are in agreement with previous studies demonstrating the renoprotective effects of ACEI/ARBs and statins (Lewis et al., 1993, 2001; Brenner et al., 2001; Lee et al., 2002; Athyros et al., 2004; Tonelli et al., 2005; Zhu et al., 2008; Ruggenenti et al., 2010).

Considering the 12-month period from baseline, the HRs of the DPP-4 inhibitors and dyslipidemia were significantly lower at eGFR decline rates of >10, >20, and >30%, indicating that DPP-4 inhibitors and dyslipidemia suppressed the eGFR decline at all thresholds examined in this study. However, dyslipidemia was reported as a risk factor for the onset of chronic kidney disease in previous studies (Muntner et al., 2000; Schaeffner et al., 2003). This contradiction may have been caused by different patient selection criteria between studies or the presence of fibrates among the treatments analyzed in this study, which was not the case in previous studies (Muntner et al., 2000; Schaeffner et al., 2003). Fibrates are used to treat dyslipidemia, and fenofibrates were reported to show renoprotective effects by significantly suppressing the progression of albuminuria in patients with hypertriglyceridemia with DM and early nephropathy, compared to placebo (Keech et al., 2005). ACEI/ARBs treatment suppressed eGFR decline (>20 and >30%), in agreement with previous studies demonstrating renoprotective effects of ACEI/ARBs (Lewis et al., 1993, 2001; Brenner et al., 2001; Zhu et al., 2008; Ruggenenti et al., 2010).

In the present study, the outcome analyzed revealed an eGFR decline (>10, >20, and >30%) over 12 months. Doubling of serum creatinine levels and the corresponding eGFR decline of >57% are hard endpoints for evaluating kidney function; however, long-term observation is necessary (Coresh et al., 2014). A mild decrease in eGFR (decline >30% over 2 years) is reported to be a useful prognostic index (Inker et al., 2013, 2014; Coresh et al., 2014). Furthermore, Coresh et al. (2014) reported that in a group with an eGFR <60%, the HR of ESRD onset was 2.4 (95% CI, 2.2–2.7) in patients whose eGFR decline was >20% over 1 year, whereas the HR was 4.0 (95% CI, 3.4–4.6) in those with a >30% eGFR decline over 1 year. In contrast, in a group with eGFR ≥60%, the HR of ESRD onset was 2.5 (95% CI, 1.8–3.3) in patients whose eGFR decline was >20% over 1 year, whereas the HR was 5.5 (95% CI, 3.6–8.4) in those with a >30% of eGFR decline over 1 year (Inker et al., 2013, 2014; Coresh et al., 2014). eGFR declines of >20 and >30% over 12 months were considered to be reasonable outcomes for predicting renal function in this study. However, the study by Coresh et al. (2014) did not include data on the eGFR decline of ≤20% at the point < 1-year period. In this study, no significant difference was observed when DPP-4 inhibitors were used at eGFR decline rates >10 and >30% in multiple logistic regression analysis; however, significant differences were found using the Cox proportional-hazards regression analysis at all eGFR decline thresholds analyzed. This could have been caused by the large number of qualifying patients in the Cox proportional-hazards regression analysis, whereas in the multiple logistic regression analysis only 55 patients presented an eGFR decline >30% after 12 months, which might cause no significant difference in the multiple logistic regression analysis. Because both regression analyses would require a larger number in each group for an accurate interpretation, we focused on eGFR decline >20%. DPP-4 inhibitors, according to both analyses, reduced the risk of eGFR decline >20%, suggesting that DPP-4 inhibitors show renoprotective effects. The Cox proportional-hazards regression analysis was conducted at baseline and after 3, 6, and 12 months; however, the accuracy improved when we focused on data across 12 months.

The results of the comparison of concurrent patient medications showed several differences. However, there were not enough patients administered antihyperglycemic agents to perform an analysis using types of antihyperglycemic agents as independent variables. A few studies have shown the renoprotective effect of antihyperglycemic agents except for incretin-related drugs, and these agents were not included in nephrotoxic agents (Perazella, 2009). Therefore, the result of renoprotective effect of DPP-4 inhibitors was robust.

To investigate the change in blood glucose levels between the groups, we compared the HbA1c decline rate 12 months from baseline for the 787 patients who had available data on HbA1c and serum creatinine levels. No significant difference was observed between the DPP-4 inhibitor-untreated (9.8%) and treated patients (9.2%, *P* = 0.538). Although we noted that the number of patients analyzed differed between the two groups, no difference in HbA1c decline rate was observed between the two groups. However, the renal function was improved in the DPP-4 inhibitor-treated group, suggesting that the DPP-4 inhibitors showed renoprotective effects independent of their antihyperglycemic effects.

The beneficial effects of coadministering DPP-4 inhibitors with renin-angiotensin-aldosterone system inhibitors have been previously reported (Groop et al., 2013; Fujita et al., 2014); however, this study suggests that DPP-4 inhibitors also possess independent renoprotective effects. A limitation of this study is that the distribution of DPP-4 inhibitors analyzed in this study was dominated by sitagliptin, possibly because of the different market release periods of the prescribed drugs.

Reduced eGFR and albuminuria in patients with DM were reported as predictors of coronary vascular death or renal outcomes (Monseu et al., 2015; Tanaka et al., 2015). However, in this study, albuminuria was not analyzed because the available data were insufficient (infrequent measurements).

In patients with end-stage cancer or those who are frail, serum creatinine levels decreased, and eGFR may be overestimated. Additionally, patients with early nephropathy may present with hyperfiltration with high apparent eGFR values because of compensatory kidney function. As this study was a retrospective study and actual patient conditions and detailed patient medical records were not available, serum creatinine values were used without correction. With low serum creatinine values, the accuracy of eGFR is reduced, potentially introducing substantial errors.

Taken together, the findings of this study suggest that DPP-4 inhibitors suppressed eGFR decrease in patients with type 2 DM and showed renoprotective effects. DM negatively affected renal function, causing irreversible deterioration. Antihyperglycemic and renoprotective effects make DPP-4 inhibitors a useful addition to DM treatments, suppressing the onset and progression of diabetic nephropathy; however, further prospective studies are necessary.

## Author contributions

All authors contributed to the study design. All authors participated in collecting and interpreting the data. HE analyzed data and drafted the manuscript. TT confirmed the analyzed data and revised the manuscript. All authors reviewed and approved the final manuscript.

### Conflict of interest statement

The authors declare that the research was conducted in the absence of any commercial or financial relationships that could be construed as a potential conflict of interest.
